# Muscle Work and Its Relationship with *ACE* and *ACTN3* Polymorphisms Are Associated with the Improvement of Explosive Strength

**DOI:** 10.3390/genes12081177

**Published:** 2021-07-29

**Authors:** Alberto Melián Ortiz, Sofía Laguarta-Val, David Varillas-Delgado

**Affiliations:** 1 Department of Physical Therapy, FREMAP-Majadahonda Hospital, 28222 Madrid, Spain; amelianor@upsa.es; 2 Department of Health Sciences, Faculty of Nursing and Physical Therapy Salus Informorum, Pontifical University of Salamanca, 37007 Madrid, Spain; 3 Department of Physiotherapy, Occupational Therapy, Rehabilitation and Physical Medicine, Faculty of Health Sciences, Rey Juan Carlos University, Alcorcón, 28922 Madrid, Spain; 4 Department of Sports Sciences, Faculty of Health Sciences, Universidad Francisco de Vitoria, Pozuelo de Alarcon, 28223 Madrid, Spain; david.varillas@ufv.es

**Keywords:** explosive strength, athletic performance, *ACE* polymorphism, *ACTN3* polymorphism

## Abstract

Background: The potential influence of genetics in athletic performance allows the search for genetic profiles associated with muscular work for the orientation of strength training and sports selection. The purpose of the study was to analyze four muscular exercises for effectiveness in improving explosive strength variables, associated to the genetics in Angiotensin Converting Enzyme (*ACE*) and α-actinin-3 (*ACTN3*) polymorphisms. Methods: A randomized controlled trial was conducted on a sample of 80 subjects allocated into four groups: concentric muscle work (CMW), eccentric muscle work (EMW), concentric-eccentric muscle (C-EMW) work and isometric muscular work (IMW), by block and gender randomization. Vertical jump, long jump, power jump, and speed were measured to study explosive strength. Genotypic frequencies of *ACE* (rs4646994) and *ACTN3* (rs1815739) were obtained by polymerase chain reaction. Results: *ACE* gen showed significant improvements regarding the DD genotype in the Sargent test (*p* = 0.003) and sprint velocity test (*p* = 0.017). In the *ACTN3* gene, the RR variable obtained improvement results with regard to RX and XX variables in long jump (*p* < 0.001), Sargent test (*p* < 0.001) and power jump (*p* = 0.004). Conclusions: The selected genes demonstrated an influence on the muscle work and the improvement in explosive strength variables with a decisive role regarding the type of muscle work performed.

## 1. Introduction

Muscle strength training is considered as an essential activity for ensuring adequate physical performance applied to any sport. Within its scope, explosive strength is the most relevant, both in team and individual sports [1]. For its development, four important aspects are considered: the type of muscle contraction and how to work it, technological support, nutrition, and lastly, the athlete’s genetic makeup [2].

Since the discovery of the human genome, considerable research has been conducted on the possible influence of genetics on athletic performance; indeed, genetic factors are believed to determine between 20% and 80% of a wide variety of traits relevant to sports performance, such as oxygen consumption, cardiac output, glucose/lipids metabolism or the relative proportion of slow-twitch and fast-twitch fibers in the trained skeletal muscle [3,4]. This previous knowledge has led research to focus on the search for genetic profiles associated with that performance and to allow, in turn, training orientation and sport selection [5,6,7].

There are three key points to understanding the athletic performance genetic variables, which make the study of deoxyribonucleic acid (DNA) an important point to have in mind, in the type of athletes training. There is mounting evidence that highlights the general idea that genes are responsible for some of the variations in athletic performance [8]. In fact, genetics would help to explain why a few athletes around the world have managed to break the 10 s barrier in the 100 m-sprint [9]. Despite the evident role that genetics plays in physical performance, there is little evidence that supports that a specific genetic variant has an important role in phenotype, at least within the range of normal distribution of genetic traits. This is probably due to the fact that complex traits are polygenic [8].

There is an increasing interest for the athletic performance genetic basis in line with the development of new and accessible methods of DNA genotype. All of this is well documented in the yearly update gene map for performance phenotypes that was published for the first time in 2001 [10].

Polymorphisms conditioning explosive strength are not the same than those of resistance. Two of the most studied polymorphisms in terms of their influence on athletic performance are the Angiotensin Converting Enzyme (*ACE*) Insertion/Deletion (I/D) allele (rs4646994). In several studies, *ACE* I/D has shown the relationship between this polymorphism and elite athlete status. In Caucasian populations, the I allele has previously been reported to be associated with enhanced elite endurance performance in long-distance runners and rowers, and with enhanced performance at high altitude [11,12,13,14]. The D allele, has been reported to be associated with strength/power sports, such as sprinting [15] and swimming events of ≤400 m [16,17,18]. On the other hand, α-actinin-3 (*ACTN3*) encodes α-actinin-3, an actin-binding protein with a structural role at the sarcomeric Z-line in glycolytic (type II, fast-twitch) muscle fibers and an increasingly evident role in the regulation of muscle metabolism [19]. The α-actinin-3 (*ACTN3*) c.1747C > T (p.R577X) (rs1815739) polymorphism has been reported in several studies in associations related to physical performance both in elite athletes and in the general population, with the 577R allele being associated with increased sprint performance [3,20,21,22], while the 577XX variant has been reported to be found at a reduced frequency in sprinters and other sprint/power athletes [3,19,23].

Both *ACE* and *ACTN3* polymorphisms can be involved in many physiological processes such as sporting performance, fatigue resistance in response to physical training, cardiac growth response, differences in muscle efficiency and strength, hypoxic ventilatory drive and skeletal muscle fiber distribution [12,24,25], enhancing physical performance improvement in power-based sports [26,27,28]. Therefore, these polymorphisms in *ACE* and *ACTN3* genes have been generally described as “genes that determine athletic performance”, with potential effects on muscle performance [29].

The aim of the study was to analyze which type of muscle work traditionally used in athletic performance is more effective in improving some explosive strength variables, as well as to try and establish if the improvement obtained is exclusively due to constant training or to subjects’ genetics. 

## 2. Materials and Methods

### 2.1. Design

The study was designed as a gender matched-paired randomized controlled trial (NCT03973060) and conducted at the Physiotherapy Unit of the FREMAP Hospital in Majadahonda (Madrid). 

### 2.2. Subjects

The sample size was calculated using the EPIDAT 4.2^®^ program, on a study population of 140 subjects. To achieve 5% accuracy by means of a normal two-sided 95% confidence interval and a power of test of 0.8, it was necessary to include 20 subjects per group in the study (80 subjects total). This sample was divided into four groups (concentric muscle work (CMW), eccentric muscle work EMW), concentric-eccentric muscle work (C-EMW) and isometric muscle work (IMW)), by blocks and gender randomization, in Excel software. 

Inclusion criteria: subjects with no musculoskeletal injuries in the lower body at the time of the study, those who showed a serious commitment to perform the entire muscle training, and who were not undergoing any other training program that could interfere with the results obtained from the measurement of the study variables. Exclusion criteria: hypertensive subjects or those whose blood pressure was not controlled at the time of the study, as well as immunosuppressed subjects.

The study was approved by the Committee of Institutional Ethics of the University Rey Juan Carlos (Madrid) (protocol code RN:181120155115) and complied with the Declaration of Helsinki for Human Research of 1974 (last modified in 2003). Participants’ rights and confidentiality were protected during the whole experiment, and the genetic information was used only for the purposes included in this investigation.

### 2.3. Muscle Work

To perform the dynamic muscle work (CMW, EMW and C-EMW) a Russian belt was used in a 40° declined surface. As this is a device that allows performing half squad-declined exercises, it was possible to reproduce a motor pattern in the lower extremities, very similar to many sports actions. 

As a measuring instrument to place the knees in the starting position, the iPhone App Goniometer-Pro was used [30].

IMW was performed on a quadriceps bench.

All the study subjects had their heart rate measured. They all underwent a 5 min warm-up on a stationary bicycle, prior to performing each muscular workout.

After the warm-up, they performed 4 sets of 12 repetitions each, with one-minute rest between sets, of each one of the three different types of dynamic movements [31]. The recommendations described by Rodríguez-Rosell et al. [32] were followed for the IMW: contraction time not exceeding 6 s; a rest period of at least 20 s was required between contractions; the total time of muscle activity did not exceed 12 min.

The training had a duration of 12 days. At the end of the training, the heart rate value was taken again.

**Figure 1:**
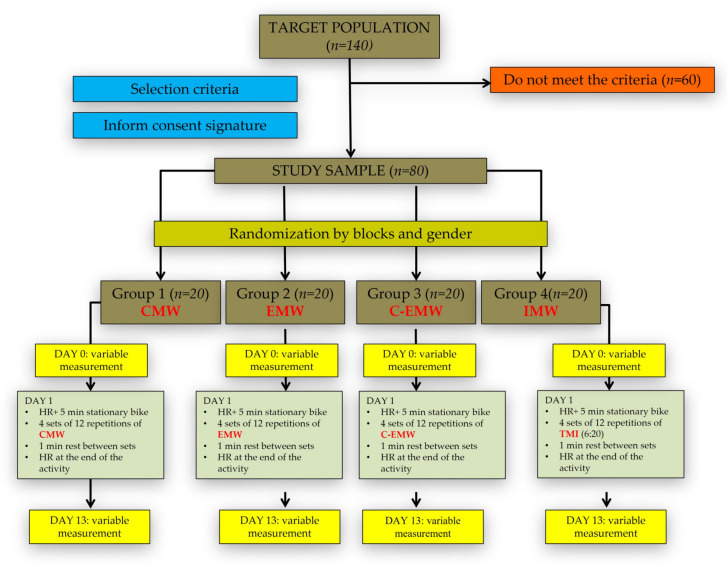
Flow chart and muscular work methodology.

The muscle strength work methodology is summarized in the flow chart shown in Figure 1.

### 2.4. Functional Test

#### 2.4.1. Long Jump (LJ)

This measurement was conducted on a smooth, horizontal surface; several marks were made on the ground leaving one meter between them. At the first mark, the subject stood with feet hip-width slightly apart. Next, a first test jump was performed; to do this, the subject was instructed that by swinging the arms and bending the knees he should jump forward as far as possible. There was a first attempt followed by a second attempt, and the second attempt’s measurement was used. As points of reference, the first mark and the heel of the foot that was further back were taken. The jump was declared a foul if the participant started the leap with any part of the foot past the first foul line.

#### 2.4.2. Sargent Test (ST)

The protocol followed in this study was the one described by Harman [33]:

1. First step: while subjects were barefoot, they stood with a smooth wall at their right side and reached up as high as possible with their right hand flat; then, the distance from the floor to the third finger of their hand (h1) was measured.
2. Second step: subjects had their third finger of the right hand marked with blue chalk dust; they stood 10 cm away from the wall and completed a countermovement jump (CMJ), with the freedom to bend the lower extremities up to a 90° angle and moving the arms, to then leap vertically as high as possible. Afterwards, a new distance was measured. This jump was performed three times, with a 45 s rest between jumps; the average of the three jumps was calculated (h2).
3. The value of the vertical jump (h) was h1−h2.

#### 2.4.3. Power Jump

To conduct this measurement, the Sayers equation was chosen, since this is the one that provides similar power values to those directly obtained by force platforms [34].

[(51.9 × height CMJ (cm)) + (48.9 × body mass (kg)) − 2007]

#### 2.4.4. Sprint Velocity Test (SV)

On a sand surface free of obstacles, a distance of 60 m was marked, and the measurement was obtained using a stopwatch. 

### 2.5. Genetic Analyses

The protocol Reliaprep™ Blood gDNA Miniprep System (Promega, Madison, WI, USA), was used following the manufacturer’s instructions, and the extracted DNA was stored at −20° until use.

The Polymerase Chain Reaction (PCR) was conducted using the method developed by Mullis [35], which specifically amplifies a single DNA molecule.

For the *ACE* gene polymorphism, the primer 5′-CTGGAGACCACTCCCATCCTTTCT-3′ and reverse primer 5′-GATGTGGCCATCACATTCGGTCAGA-3′ were used. When homozygous individuals were observed for the D allele (D/D), we performed a second round of amplification to avoid mistyping produced by the D allele that prevents the existence of non-amplified allele I, using forward primer 5′-TGGGACAGCGCCCGCCACTAC-3′ and reverse primer 5′-TCGCCAGCCCTCCCATGCCCATAA-3′. 

For the polymorphism used in the *ACTN3* gene, the primer 5′-CGCCCTTCAACAACTGGCTGG-3′ and reverse primer 5′-GGGTGATGTAGGGATTGGTGGAG-3′ were used.

Results of SNPs were analyzed by 2% agarose gel.

### 2.6. Statistical Analyses

The statistical analyses were calculated using Statistical Package for the Social Sciences (SPSS), v.21.0 for Windows (IBM Corp. Released 2012. IBM SPSS Statistics for Windows, Version 21.0. Armonk, NY: IBM Corp). The descriptive analysis was assessed in means and standard deviations (SD) for quantitative variables, while qualitative variables were expressed as frequencies and percentages. The Hardy–Weinberg equilibrium (HWE) was tested for each polymorphism using χ^2^ tests. T test and one-way ANOVA were used for the comparison of the different functional tests between groups, with an F-value estimate. The improvement of intervention test variables was calculated with confidence intervals of 95% (95% CI) for each polymorphism.

*p* < 0.05 results were considered statistically significant.

## 3. Results

The sample distribution for the *ACTN3* (*p* = 0.582) and *ACE* (*p* = 0.371) polymorphisms met the HWE. 

The demographic distribution of the participants at the beginning of the study is shown in Table 1.

Table 2 shows the differences between the study groups, regarding the explosive strength variables analyzed.

The frequency of the *ACE* and *ACTN3* variants in all subjects is shown in Table 3, while the distribution within the groups of study is shown in Table 4.

The *ACE* gene showed differences among the three genotypes (*p* = 0.003) in the ST, due to an improvement in 1.97 cm (95% CI; 1.179–2.422; *p* = 0.026) in the DD and ID genotypes. There was also a jump improvement of 1.46 cm (95% CI: 1.215–1.733; *p* < 0.001) between the DD and II genotypes. Regarding the SV test, the DD genotype showed improvement compared with the other two (*p* = 0.017); this was of 0.248 s (95% CI: 0.142–0.425; *p* = 0.021) between DD and ID genotypes, and of 0.295 s (95% CI: 0.172–0.483; *p* = 0.004) between DD and II genotypes (Table 5).

Regarding the *ACTN3*, there was a statistical difference between variants (*p* < 0.001) in LJ test. The XX variant showed less improvement compared with the RX in −8.03 cm (95% CI: −12.262–5.138; *p* < 0.001) and with the RR, −6.79 cm (95% CI: −9.262–4.2622; *p* < 0.001). The ST showed similar results with differences between genotypes (*p* < 0.001). The XX variant showed the worst results in terms of improvement; if compared with the RR variant, the result was −2.055 cm (95% CI: −3.251–1.163; *p* < 0.001), but there was no difference between the XX and RX variants (*p* = 0.068). In power jump, the participants showed a general improvement in the XX variant with respect to the other variants (*p* = 0.004), which confirms a difference of 183.044 (95% CI: 73.373–367.262; *p* < 0.001) when compared with the RR variant, and of 183.744 (95% CI: 82.274–401.632; *p* < 0.001) when compared with the RX variant. There were no differences shown between genotypes in the SV (*p* = 0.174) (Table 6).

However, among the different groups of study, neither the *ACE* nor *ACTN3* polymorphisms showed differences in the improvement of functional tests (Table 7).

## 4. Discussion

In athletic performance in healthy subjects while performing different muscular exercises the influence of two types of conditioning factors have been identified: external or environmental (motivation, type of muscle training, body weight, diet, etc.) and others internal to the athlete himself, such as genetic factors [36]. This recently published study tried to demonstrate which of the selected muscular exercises was more effective in improving some performance variables of explosive strength [37]. The aim was to establish whether this improvement was due to the type of muscle work or to the genetics of the subjects under study. For this purpose, we focused on two genes that play a key role in the different physiological processes of power sports training, which directly or indirectly may be related to sport and muscle performance: *ACE* and *ACTN3* [13,22,38].

The ability of skeletal muscles to produce force at a high velocity is crucial for success in power and sprint performance and is strongly influenced by genetics. Without the appropriate genetic make-up, an individual reduces his/her possibilities of becoming an exceptional power or sprinter athlete [39]. *ACTN3* and *ACE* genes were found to be related in this regard, by showing that *ACE* I and *ACTN3* X alleles determine speed and power for Lithuanian athletes [24]. This data differs from the data shown in this study, as it is proven that participants with the *ACE* D allele show a power and speed improvement in LJ, but there is no association with these physiological variables in the *ACTN3* gene. Moreover, the *ACE* D allele is associated with a higher explosive strength, and an improvement in the power and speed categories previously shown [24,40,41]. 

The study subjects were not trained athletes and there was no genetic disintegration in the selection of the participants, therefore the improvement is more significant than in more trained athletes. In line with other researchers, it can be confirmed that the absence of a functional *ACTN3* in the fast-twitch muscle fibers is compensated [42,43,44]. Those subjects who have *ACE* D/D and I/D genotypes show the potential to achieve better results in power sports, an important aspect for them to use strengthening working techniques to increase power performance, based on their inborn conditions. 

The different activities performed by the study subjects do not show any influence on power improvement, as previously shown [37] in relation to *ACE* and *ACTN3* genes (Table 7). This presents contradictory data to those shown in the study of strength training, in which *ACTN3* R577X polymorphism can have an important role in the abilities related to muscle force, by providing a beneficial effect to those athletes who have the X allele [45]. Likewise, it is suggested that *ACE* and *ACTN3* polymorphisms (single or combined) exert a significant influence in the muscle phenotypes of Caucasian women in response to high-speed power training [41]. Thus, these are likely factors in modulating exercise-related phenotypes in older women, particularly in response to a power training stimulus, by adjusting power activities to the study subjects’ genotype, opening the path to future research in this field to show the association genotype/phenotype in the genes presented.

A high frequency of study subjects is heterozygous in the two genes studied. The CC and CT genotypes of the *ACTN3* gene predominate over the TT genotype in the sample subjects, which indicates that more than half of the study subjects would be benefited for an association of explosive strength activities. This fact is also confirmed by the *ACE* gene, where almost 40% of the subjects were carries of the DD genotype. The percentage of the different genotypes of the *ACTN3* gene in the Spanish population (CC 30%, CT 50%, TT 20%), if compared with those obtained in this study, are found to be similar in that CT is the most predominant genotype, which shows that half of the Spanish population would be heterozygous for this gene.

The CMW group brought together more subjects with explosive strength genotypes; however, the group with the best improvement in the results of the variables studied was the C-EMW. This suggests that this study does not demonstrate a direct genetic influence between the muscle work performed and the resulting improvement obtained in explosive strength variables, but rather that the type of muscle work performed plays a more decisive role. Furthermore, the subjects with *ACE* DD variant showed greater improvement in long jump, vertical jump, and power jump, while subjects with *ACTN3* TT genotype also improved in the same variables. In a 2010 study on non-athletic subjects, it was found that speed and vertical jump were not influenced by *ACTN3* genotype [46], which highlights the fact that the lack of association between this genotype and muscle performance in general population, suggests that this polymorphism does not identify subjects to improve their physical performance.

In 2003, Yang et al. showed that the frequency of the TT genotype had increased in male power athletes in Australia compared with the controls; meanwhile, none of the female Olympian power athletes had this genotype [3]. These findings are supported by independent case and control studies conducted in Greek elite athletes [47], elite-level strength athletes in United States [48], Russian power athletes [49], Israeli sprinters [26] and Japanese male athletes [50].

As observed, despite the differences in some studies, most authors state that CC genotype is associated with power and speed physical performance, while TT genotypes associate with endurance performance. However, it should also be taken into consideration that most studies are conducted at European level, and that these results would widely differ if they were drawn from African or Asian athlete population. 

Recently, findings have been published that prove the potential influence of *ACTN3* and *ACE* polymorphisms in dynamic isometric strength tests [51], which could be used to suggest hypothesis about the known effects of genetics in different muscular work in power-based sports.

This study presents some limitations. The fact that, although there were 80 subjects, due to having four study groups, these groups only had 20 subjects. Similarly, having a fifth control group, which had not performed any kind of muscle training, would have given more validity to the results. 

Once the potential influence of genetics on athletic performance and muscle function has been determined, this information could be very useful for establishing the approach to sports for young athletes involved in sport talent programs.

## 5. Conclusions

About half of the participants of the study were heterozygous for the two candidate genes studied, which suggests that, for the most part, the subjects would benefit from explosive strength activities. The CMW group brought together more subjects with explosive strength genotypes; however, the group with the best improvement in the results of the variables studied was the C-EMW. This suggests that this study does not demonstrate influence between the muscle work performed and the improvement obtained in explosive strength variables. Furthermore, the subjects with the *ACE* DD genotype showed greater improvements in long jump, vertical jump and power jump, while subjects with the *ACTN3* CC genotype (prevalence of fast-twitch fibers) also improved in the same variables. 

Finally, in this study, the candidate genes analyzed demonstrated an influence on the muscle work and the improvement obtained in explosive strength variables with a decisive role regarding the type of muscle work performed.

## Figures and Tables

**Table 1:** Demographic characteristics of subjects.

Demographics *	CMW (*n* = 20)	EMW (*n* = 20)	C-EMW (*n* = 20)	IMW (*n* = 20)	^†^*p-*Value
Sex					-
Male	10 (50.0%)	10 (50.0%)	10 (50.0%)	10 (50.0%)	
Female	10 (50.0%)	10 (50.0%)	10 (50.0%)	10 (50.0%)	
Age	26.74 (2.29)	22.58 (3.45)	28.05 (9.03)	22.35 (3.15)	0.081
Body height (cm)	170.1 (7.32)	169.0 (7.52)	169.3 (6.77)	171.8 (11.16)	0.717
Body weight (kg)	68.13 (10.87)	67.18 (12.52)	66.29 (10.63)	68.03 (14.05)	0.961
Blood pressure (mmHg)	121.5 (10.54)	128.8 (18.16)	125.3 (14.99)	125.9 (13.56)	0.485
Heart rate (bpm)	75.16 (19.78)	77.32 (14.50)	76.55 (14.18)	79.20 (13.11)	0.876

bpm, beats per minute; cm, centimetres; CMW, concentric muscle work; C-EMW, concentric-eccentric muscle work; EMW, eccentric muscle work; IMW, isometric muscle work; Kg, kilograms; mmHg, millimetres of mercury. * Data are presented as mean and SD for continuous variables and as frequency (%) for categorical variables. ^†^ *p-*Value calculated for the professional vs. nonprofessional comparison by using the Fisher’s exact test (categorical variables) and Student’s *t* test (continuous variables).

**Table 2:** Comparative of functional test in study groups.

	LJ	ST	SV	Power Jump
**CMW vs. EMW**	*p* = 0.819	*p* = 0.045	*p* = 1.000	*p* = 0.040
**CMW vs. C-EMW**	*p* = 0.103	*p* = 0.647	*p* = 0.923	*p* = 0.305
**CMW vs. IMW**	*p* = 1.000	*p* = 0.207	*p* = 0.344	*p* = 0.210
**EMW vs. C-EMW**	*p* = 0.080	*p* = 0.003	*p* = 0.887	*p* < 0.001
**EMW vs. IMW**	*p* = 0.809	*p* = 0.620	*p* = 0.324	*p* = 0.256
**C-EMW vs. IMW**	*p* = 0.030	*p* = 0.176	*p* = 0.234	*p* = 0.089

CMW, concentric muscle work; C-EMW, concentric-eccentric muscle work; EMW, eccentric muscle work; IMW, isometric muscle work; LJ, long jump; ST, Sargent test; SV, sprint velocity test.

**Table 3:** Frequency of *ACE* and *ACTN3* variants in study subjects.

*ACE*	Subjects (*n* = 80)
II	10 (12.5%)
ID	39 (48.8%)
DD	31 (38.8%)
***ACTN3***	**Subjects (*n* = 80)**
RR	33 (41.3%)
RX	36 (45.0%)
XX	11 (13.8%)

**Table 4:** Distribution of *ACE* and *ACTN3* variants in the study groups.

*ACE*	CMW (*n* = 20)	EMW (*n* = 20)	C-EMW (*n* = 20)	IMW (*n* = 20)	*p* Value
II	1 (5.0%)	3 (15.0%)	4 (20.0%)	2 (10.0%)	
ID	10 (50.0%)	9 (45.0%)	10 (50.0%)	10 (50.0%)	0.847
DD	9 (45.0%)	8 (40.0%)	6 (30.0%)	8 (40.0%)	
***ACTN3***	**CMW (*n* = 20)**	**EMW (*n* = 20)**	**C-EMW (*n* = 20)**	**IMW (*n* = 20)**	
RR	13 (65.0%)	5 (25.0%)	6 (30.0%)	9 (45.0%)	
RX	5 (25.0%)	11 (55.0%)	11 (55.0%)	9 (45.0%)	0.213
XX	2 (10.0%)	4 (20.0%)	3 (15.0%)	2 (10.0%)	

CMW, concentric muscle work; C-EMW, concentric-eccentric muscle Work; EMW, eccentric muscle work; IMW, isometric muscle work.

**Table 5:** Improvement in *ACE* variants regarding functional test.

	*ACE*	Units Won *	*p* Value
	II	8.16 (1.76)	
LJ (cm)	ID	8.68 (2.163)	0.278
	DD	6.70 (1.943)	
	II	0.606 (0.085)	
ST (cm)	ID	0.792 (0.157)	0.003 ^†^
	DD	1.762 (0.363)	
	II	58.119 (10.521)	
Power jump	ID	95.888 (21.624)	0.217
	DD	83.787 (12.216)	
	II	0.219 (0.099)	
SV (s)	ID	0.266 (0.068)	0.017 ^†^
	DD	0.514 (0.062)	

LJ, long jump; ST, Sargent test; SV, sprint velocity test. * Units won between baseline to end intervention. ^†^ Statistical significance was set at *p* < 0.05.

**Table 6:** Improvement in *ACTN3* variants regarding functional test.

	*ACTN3*	Units Won *	*p* Value
	RR	8.61 (1.01)	
LJ (cm)	RX	9.85 (1.748)	<0.001 ^†^
	XX	1.82 (2.263)	
	RR	2.042 (0.322)	
ST (cm)	RX	0.676 (0.127)	<0.001 ^†^
	XX	−0.013 (0.026)	
	RR	111.35 (23.263)	
Power jump	RX	112.02 (18.842)	0.004 ^†^
	XX	−71.694 (15.842)	
	RR	0.363 (0.080)	
SV (s)	RX	0.385 (0.093)	0.174
	XX	0.239 (0.088)	

LJ, long jump; ST, Sargent test; SV, sprint velocity test. * Units won between baseline to end intervention. ^†^ Statistical significance was set at *p* < 0.05.

**Table 7:** Improvement differences between *ACE* and *ACTN3* variants on different groups.

	*ACE*	
	F	*p* Value
LJ (cm)	1.317	0.192
ST (cm)	0.716	0.800
Power jump	1.357	0.518
SV (s)	0.727	0.825
	***ACTN3***	
	**F**	***p* value **
LJ (cm)	0.984	0.513
ST (cm)	0.641	0.863
Power jump	1.453	0.495
SV (s)	1.486	0.170

LJ, long jump; ST, Sargent test; SV, sprint velocity test.

## Data Availability

Not applicable.
